# Understanding Parental Satisfaction in Caring for Children with Cerebral Palsy

**DOI:** 10.3390/healthcare13020110

**Published:** 2025-01-08

**Authors:** Aleksandra Kołacka, Maja Matthews-Kozancecka, Oskar Komisarek, Jacek Kwiatkowski, Aleksandra Domagalska, Włodzimierz Samborski, Ewa Mojs, Mirosław Andrusiewicz, Roksana Malak

**Affiliations:** 1 Department and Clinic of Rheumatology, Rehabilitation and Internal Medicine, Poznan University of Medical Sciences, 61-545 Poznań, Poland; 2 Department of Social Sciences and the Humanities, Poznan University of Medical Sciences, 60-806 Poznań, Poland; 3 Department of Otolaryngology, Audiology and Phoniatrics, Collegium Medicum, Nicolaus Copernicus University in Torun, Jagiellońska 13-15, 85-067 Bydgoszcz, Poland; 4 Faculty of Medicine, Poznan University of Medical Sciences, 60-812 Poznań, Poland; ola.sledzinska1@gmail.com; 5 Department of Clinical Psychology, Poznan University of Medical Sciences, 60-812 Poznań, Poland; 6 Department of Cell Biology, Poznan University of Medical Sciences, 60-806 Poznań, Poland; andrus@ump.edu.pl

**Keywords:** cerebral palsy, parents’ life satisfaction, sten scores

## Abstract

Introduction: The life satisfaction of parents of children with cerebral palsy should be assessed. Parenting a child with a disability may bring more challenges and efforts, impacting overall quality of life. Aim of the Study: the study aimed to evaluate the satisfaction with life of parents of children with cerebral palsy. Material and Methods: The study was designed to assess the satisfaction with life of parents of children with cerebral palsy. To measure this outcome, we developed an original survey consisting of 29 questions inspired by the Satisfaction With Life Scale (SWLS). The study involved 43 parents or legal guardians of children diagnosed with cerebral palsy. Results: A correlation was identified between parental life satisfaction and the amount of leisure time reported by parents (*p* = 0.004, *R* = 0.46). The research indicates that parental life satisfaction does not depend on the parent’s satisfaction with the level of therapy *(p* > 0.05) and the degree of improvement in the child’s functioning (*p* > 0.05). Conclusions: The level of parental life satisfaction does not depend on the level and outcome of therapy. The more leisure time the parents have, the greater their life satisfaction is. Parents of children with cerebral palsy should take care of their free time.

## 1. Introduction

Parenthood is a journey when moments of joy and challenges significantly shape parents’ and children’s lives. Each child needs parents and caregivers. Therefore, the emphasis on caring for a child should also be placed on the parents. Empirical investigations have verified a link between parenting practices, self-esteem, and overall life satisfaction [1]. Not only do children need empowerment, but parents also need it. Nurturing a child with cerebral palsy adds a layer of complexity to parental responsibilities, potentially exerting a detrimental impact on their overall quality of life. The implications of these caregiving duties are profound, necessitating parents to adeptly navigate a dynamic landscape of evolving requirements tailored to their child’s unique needs [2].

It is well-known that “a happy parent, a happy child”. Even if a child is diagnosed with developmental dysfunctions, the focus should be placed not only on the child but also on the parents. Children’s skills and quality of life are usually evaluated. However, parental conditions, such as their satisfaction with life, should also be considered. A new person, a new child, is a big change for each family member. It seems to be much more challenging for families if a child brings a diagnosis referring to their condition, for instance, cerebral palsy.

Cerebral palsy (CP) is a group of non-progressive dysfunctions resulting from central nervous system disorders that affect a child’s motor development [3]. Cerebral palsy manifests at a prevalence rate of 2.11 per 1000 live births in a pooled children population, underscoring its significance within the landscape of pediatric neurodevelopmental disorders [4]. European data indicates that the average occurrence of CP stands at 2.08 per 1000 live births. However, in infants weighing below 1500 g at birth, the incidence rate is 70 times greater than those weighing over 2500 g [5,6]. The etiology of this condition is multifaceted, encompassing a spectrum of factors operative during prenatal, perinatal, and postnatal phases. Preeminent among these determinants are instances of premature birth, fetal hypoxia and ischemia, extremely low birth weight, multiple gestations, gestational infections, and maternal age falling below 16 years or exceeding 40 years [7]. Children diagnosed with cerebral palsy form a notably heterogeneous cohort characterized by variability in the onset and severity of symptoms. Indications suggestive of cerebral palsy encompass a spectrum of manifestations, including but not limited to spastic, ataxic, hypotonic, dyskinetic or dystonic paresis, involuntary movements, disruptions in motor coordination, challenges in maintaining balance, developmental aberrations, and very often but not always instances of epilepsy [8]. Following Ingram’s classification, cerebral palsy in children is discerned across six distinct forms: bilateral spastic paralysis (diplegia), spastic hemiparesis (hemiplegia), bilateral hemiparesis (bilateral hemiplegia), cerebellar form (ataxia), extrapyramidal form (dyskinesia), and mixed forms [9,10].

In contexts characterized by limited economic resources, caregivers of children with cerebral palsy encounter specific challenges that demand targeted interventions to enhance the quality of life for both caregivers and their wards [10,11].

To summarize, the impact of CP on parental satisfaction can be caused by fear and anxiety for the future of a child. Uncertainty of the outcome of rehabilitation and efforts that children and parents put into everyday life may also change life satisfaction. Time and expenses that in typical parenthood are allocated to vacations are often spent on orthopedic aids or therapies.

The presented research aimed to evaluate the view of life satisfaction of parents in the context of caring for a child with cerebral palsy. Moreover, the measure of life satisfaction was not linked to the type of cerebral palsy.

### Hypothesis

The following hypotheses were established for the purposes of this study:

1. The level of parental life satisfaction depends on the level of satisfaction with therapy.
2. The level of parental life satisfaction depends on improving the child’s functioning.
3. The level of parental life satisfaction depends on how much free time the parent has.
4. The level of parental life satisfaction depends on the time spent caring for the child.

## 2. Materials and Methods

The study was conducted between June 2022 and July 2022 among caregivers of children diagnosed with cerebral palsy. The study was conducted according to the guidelines of the Declaration of Helsinki and approved by the Institutional Review Board of Poznan University of Medical Sciences (No. 481/21).

### 2.1. Participants

We invited parents of children with cerebral palsy who attend the daily care unit of rehabilitation in the Rehabilitation Center of Great Poland.

The inclusion criteria were as follows:

- parental consent to complete the survey;
- confirmed diagnosis of cerebral palsy of the child;
- evaluated Gross Motor Function Classification System (GMFCS) level [12,13].

The exclusion criteria included the following:

- lack of parental consent to provide answers;
- lack of a characteristic diagnosis of neurological palsy in the child;
- unspecified level of functioning according to the GMFCS scale.

The study involved 43 legal guardians, of whom, after applying inclusion and exclusion criteria, 38 individuals (1 man and 37 women) qualified for the study.

The characteristics of the group in terms of place of residence are presented in Table 1. The percentage values given were given after rounding them to one decimal place.

### 2.2. Instruments

The Satisfaction With Life Scale (SWLS) was an inspiration for the creation of the original survey, which consisted of 29 questions [14]. The Satisfaction With Life Scale (SWLS) contains five sentences to which participants can respond as follows [14]:

7—I strongly agree;

6—I agree;

5—I somewhat agree;

4—I neither agree nor disagree;

3—I somewhat disagree;

2—I disagree;

1—I strongly disagree.

Each question was designed to elicit fundamental information concerning the parent or caregiver’s life satisfaction. The study focused on analyzing relationships between variables rather than validating the internal consistency of a scale. Cronbach’s alpha was not calculated due to the small sample size, which could lead to unreliable estimates. What is more, the questionnaire included items assessing diverse constructs, making Cronbach’s alpha unsuitable for this analysis. We asked some questions, which we directed to all parents, to assess their satisfaction with life. An example of this is question number 28:

Do you have free time, reserved exclusively for yourself?

- yes, on average, once a week;
- yes, 2–3 times a month;
- yes, but irregularly;
- I don’t have such opportunities.

We also took into account age, the sociodemographic details of each parent, and the child’s comorbidities [12,13]. Additionally, we inquired into details regarding the child’s rehabilitation. The questions, structured as closed-ended, offered respondents the flexibility of providing singular or multiple responses. The questionnaire was divided into parts. The first part pertained to basic sociodemographic data on legal guardians and children. The second part involved an assessment of the child’s functioning. The third part evaluated the parent’s satisfaction with the child’s therapy.

The participants’ anonymity was preserved to uphold ethical standards, and their involvement was voluntary. The surveys were carried out through two collection strategies: traditional PAPI (Paper and Pen Interview) and CAWI (Computer-Assisted Web Interview), with the selection of format left to the discretion of the participants. The data from these questionnaires constituted the foundational dataset for the ensuing research, which is clarified in this article.

### 2.3. Statistical Analysis

Statistical analyses were performed using Statistica^®^ Version 13.5.0 software for Windows (TIBCO Software Inc., Palo Alto, CA, USA) and PQStat 1.8.0.414 software (PQStat software; Poznan, Poland). A two-sided Mann–Whitney *U* test and a Kruskal–Wallis test were used with Dunn’s post hoc test for differences assessment. A Bonferroni–Hochberg correction was used to test multiple comparisons. A Jonckheere–Terpstra test was used for ordered categorical data to determine the significance of the trends. Spearman’s rank correlation tests determined the correlation coefficient (*R*) or Kendall’s *Tau* correlation coefficient (τ) between parameters. Data were considered statistically significant at *p* < 0.05.

## 3. Results

The average age of the parents was 37 ± 8.1 years (median 37; interquartile range (31–43) years; min–max (26–64)). Fifteen participants declared they have only one child, and 23 participants have 2–4 children. The largest number of respondents (31 people) share the responsibility of caring for a child with a partner. Four people declared that they take care of a child on their own. One person uses the help of third parties (neighbor, carer, etc.). Two people refused to provide this information. A total of 10 people work in their profession; 28 people do not work professionally.

The questionnaire helped us to reveal that the level of parental life satisfaction demonstrates no statistically significant correlation between parent’s satisfaction and the level of therapy (defined as the intensity of therapy, treatment effects, the therapist’s skills and commitment, and the conditions in which the treatment was carried out; *p* = 0.756).

Similarly, the survey outcomes reveal that parental life satisfaction remains unaffected by the degree of improvement in the child’s functioning (*p* > 0.05).

**Figure 1:**
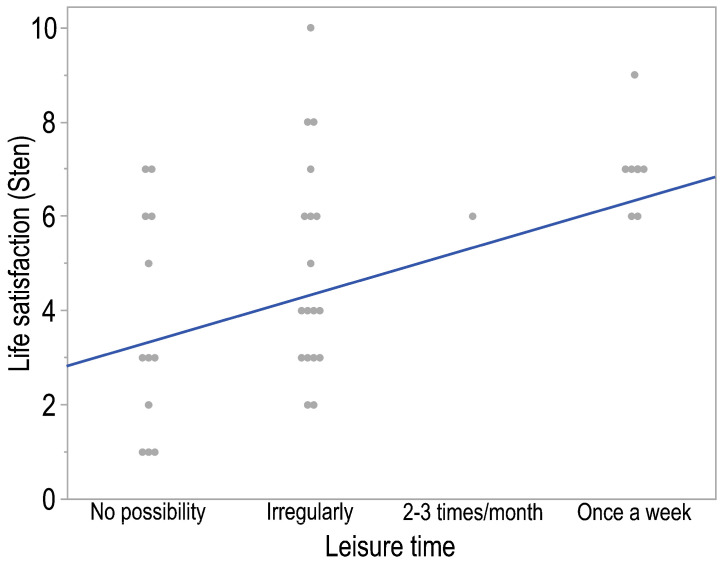
The correlation plot of life satisfaction of parents (legal guardians) and their leisure time indicates a monotonic relationship. The regression line is shown in blue.

Contrastingly, a significant, moderate positive correlation between parental life satisfaction and the amount of leisure time reported by the parent was established (*p* = 0.004, *R* = 0.46). This correlation is noteworthy for its positive nature, signifying that an increase in the self-declared amount of leisure time corresponds to a higher level of parental life satisfaction (Figure 1).

**Figure 2:**
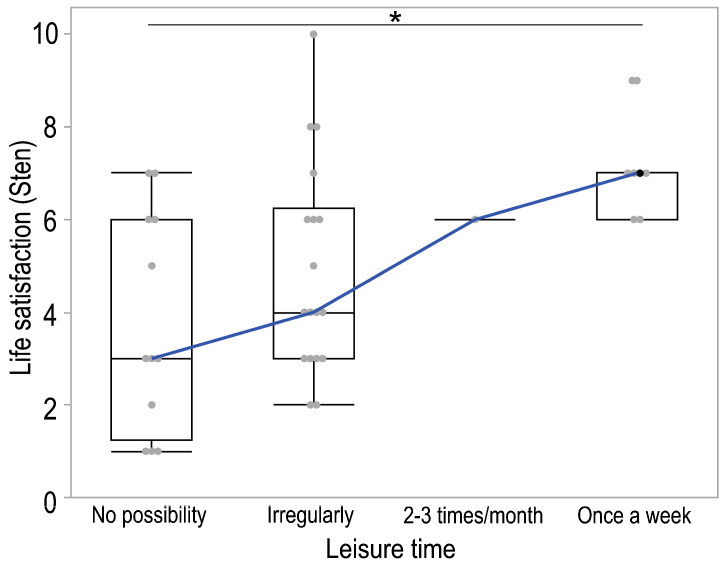
Boxplot illustrating the distributions of sten scores of parents (legal guardians) by leisure time. The box in each box plot spans the interquartile range, and lines indicate the location of the first quartile, median, and third quartile. Whiskers extend to the last data point within 1.5 times of the interquartile range in either direction, and additional points present outlier observations. The blue line represents the Jonckheere–Terpstra trend; * *p* < 0.05.

Moreover, noteworthy distinctions in the sten level, indicative of the level of life satisfaction, were observed among groups of parents based on their self-reported amount of free time (*p* = 0.017). In each instance, cumulative assessments of sentences were totaled, followed by applying a sten scale, with each result assigned a sten value according to established norms. The group lacking the opportunity for free time exhibited the lowest sten level, contrasting with the group reporting, on average, one instance of free time per week, which displayed the highest sten level (*p* = 0.018). No statistically significant differences were discerned among the remaining groups. The Jonckheere–Terpstra test also showed a significant ascending trend (*p* = 0.002) in the sten level according to leisure time (Figure 2).

These findings underscore the potential impact of free time availability on parental life satisfaction as detailed in the results presented.

**Figure 3:**
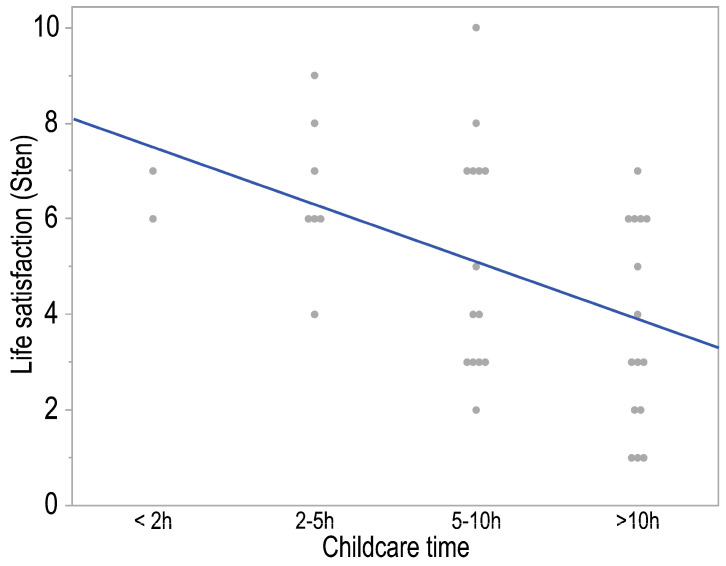
The correlation plot of life satisfaction and parents’ (legal guardians) childcare time indicates a monotonic relationship. The regression line is shown in blue.

The survey study demonstrated a significant, moderate, and inversely proportional correlation between a parent’s level of life satisfaction and the amount of time devoted to childcare (*p* = 0.002; *R* = −0.48). The shorter the time spent on childcare, the higher the parent’s level of life satisfaction (Figure 3).

**Figure 4:**
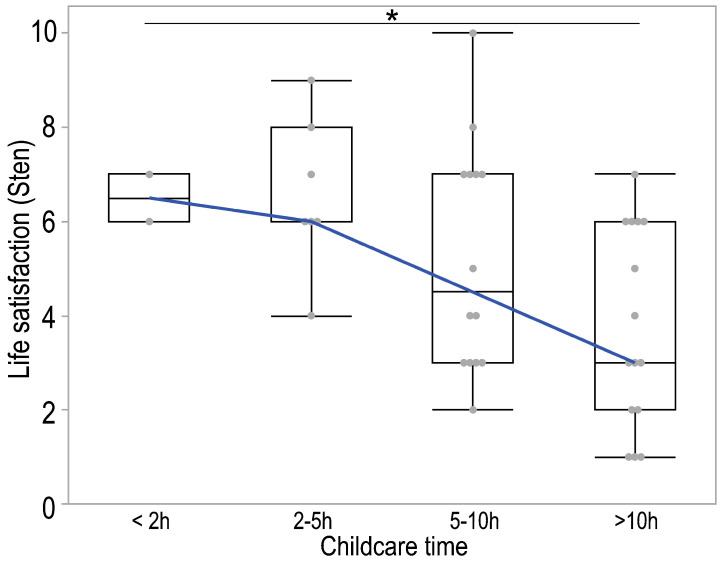
Boxplot illustrating the distributions of sten scores of parents (legal guardians) by the amount of time devoted to childcare. The box in each box plot spans the interquartile range, and lines indicate the location of the first quartile, median, and third quartile. Whiskers extend to the last data point within 1.5 times of the interquartile range in either direction. The blue line represents the Jonckheere–Terpstra trend; * *p* < 0.05.

A statistically significant difference in sten scoring was also demonstrated between groups of parents who devoted time to childcare (*p* = 0.032). The highest sten values occurred in the group of people declaring less time spent on childcare (<2 h per day and 2–5 h per day), and the lowest in the case of spending more than 10 h a day on childcare. A post hoc Dunn’s test showed significant differences between parents declaring 2–5 h of childcare and the group spending more than 10 h on it (*p* = 0.049). A Jonckheere–Terpstra test also showed a significant, descending trend (*p* = 0.003) in sten level according to childcare time (Figure 4).

Our new findings show that parents’ satisfaction with life is based on the level of their free time, not on the level of therapy.

## 4. Discussion

Cerebral palsy is characterized as a non-progressive disorder with the potential to impact motor, postural, and balance development [8]. This condition influences various facets of life, affecting the child and profoundly influencing the parent’s experiences [15,16].

Parents confronted with disabilities in children and also a diagnosis of cerebral palsy in their child undergo a substantial adaptation process, leading to significant stress [17,18]. Stress may accompany their inpatient experience [19]. Many studies show that stress is strongly associated with low life satisfaction [20,21,22,23]. By reducing parental stress, life satisfaction may improve [24].

The challenges encompass the need to rehabilitate and attend to crucial therapies, sometimes special education and equipment. Our study found that parents who devoted more time to care had lower satisfaction with life. Moreover, Pousada et al. [2] indicate in the results of their review that mothers of children with cerebral palsy are characterized by fatigue, higher levels of stress and depression, and a lower quality of life compared to parents of children without disabilities. The resultant strain often gives rise to heightened levels of fatigue, leading to frequent conflicts [25,26]. Parents of children with CP care for household matters and other children. Prajakta et al. [3] suggest that could be a possible explanation for these findings because the responsibility of caring for an adolescent with CP affects the parents’ physical and social well-being, freedom, family well-being, and financial stability. The cumulative effect of these challenges invariably impacts the parents’ overall quality of life and satisfaction levels. However, research shows that even if parents experience stress and low satisfaction with life, their well-being can improve when they receive social support [27]. What is more, the severity of disability of a child is not significantly related to maternal well-being [22].

However, many factors affect parents’ quality of life: mental and physical health, material status, and family support.

Surprisingly, our research also shows unexpected results that a parent’s life satisfaction is not dependent on the level of therapy defined as the intensity of therapy and treatment effects—including improvements in the child’s functioning, the therapist’s skills and commitment, and the conditions in which the therapy is carried out. The sten level, indicative of life satisfaction, did not significantly change based on therapy expectations within the studied parent group. Despite expectations that higher therapy satisfaction might alleviate parental stress and fatigue, our findings did not support this assumption. Similarly, a study by Tuna et al. [28], reviewed by Pousada [2], seems far from confirming the hypothesis: parental life satisfaction depends on improving the child’s functioning and satisfaction with therapy. It was found that motor ability level measured by the Gross Motor Classification System was not correlated with parents’ life satisfaction. Hamzat and Mordi [29] and Sajedi et al. [30] showed no statistically significant differences in satisfaction of life and the level of Gross Motor Function Classification System among children with CP. The actual motor ability of a child was not related to caretakers’ psychological states in a study by Ho et al. [30] or by Pousada et al. [2] either.

Notably, our study shows how important leisure time is for families, including parents. We have not found any paper that has shown this result before. More articles present the role of children’s free time [31,32]. Other researchers, for example, Prajakta et al. [3], emphasize that adolescents need to feel their family’s acceptance and valuation despite CP. The imperative for active parental participation in therapy is linked to a potential reduction in time availability for parental relaxation. Conversely, research by Wang et al. [14] suggests a negative correlation between parental stress and the extent of social support received by mothers of children with cerebral palsy, influencing the overall level of parental life satisfaction.

The presented research results underscore the necessity for additional investigations into the factors influencing parental life satisfaction. A more comprehensive examination of this topic can potentially enhance the quality of life for both the parent and child. Active participation by both the parent and child in the therapeutic process positively affects therapy outcomes and enhances the child’s functioning. The main implication of the study and analyzed literature is that the intervention should be addressed to the whole family to improve parents’ resources and help them cope better with daily caring tasks [33].

Monitoring subjective life satisfaction and well-being provides valuable insights for policymakers and governments. This information can be instrumental in assessing the positive outcomes of public assistance programs and fostering societal improvements beyond economic development. Life satisfaction as a part of well-being encompasses individuals’ assessments of their lives, incorporating positive emotions, engagement, life satisfaction, and a sense of purpose. Importantly, substantial empirical evidence highlights that high levels of life satisfaction and well-being contribute positively to health and longevity, workplace productivity, and the quality of social relationships [34].

Comparative analyses of therapies employed in the rehabilitation of children with cerebral palsy and corresponding observations by parents can offer valuable insights for tailoring rehabilitation processes to meet the needs and capabilities of both the child and parent. Additionally, further investigation is needed to explore the impact of the child’s desire to attend therapy sessions on their engagement in the therapeutic process and the efficacy of exercises. Further research can contribute to a more nuanced understanding of the dynamics involved.

### Limitation of the Study

Limitations of the study include a relatively small group of children with cerebral palsy and their legal guardians. There was no control group because intensive rehabilitation, which is part of everyday life, makes the research group very specific and homogenous. We acknowledge the limitation that causal inferences cannot be drawn from the findings of our study. As our research is correlational, it captures associations rather than establishing direct cause-and-effect relationships. This limitation is inherent to the methodology employed and underscores the need for future research employing experimental or longitudinal designs to explore potential causal pathways

## 5. Conclusions

It seems that each person is looking for happiness, satisfaction with life, and well-being. The research findings indicate that parental life satisfaction remains independent of satisfaction with therapy or improving the child’s functioning. Moreover, no correlation was observed between the child’s desire to attend therapy and parental life satisfaction. However, a positive correlation was noted between the amount of free time the parent has and their life satisfaction. A negative correlation was also identified; the less time the parent spends caring for the child, the higher their life satisfaction. Interestingly, the child’s desire to attend therapy is not contingent on the amount of treatment they have. A combination of additional time for personal needs and a robust support system can significantly contribute to parents’ well-being and the quality of care they provide to their children. Our main conclusion refers to parental need for their free time. We can suspect that very often parents of children with challenges such as CP may be so devoted to their children that they can forget about themselves. They should gain gold medals for their everyday life. However, happy parents mean happy children.

## Figures and Tables

**Table 1:** Presentation of the number of participants depending on the place of residence.

Place of Living	N	Percentage Share of Responses
Village	14	36.8%
City up to 50,000 inhabitants	10	26.3%
City from 50,000 to 100,000 inhabitants	3	7.9%
City from 100,000 to 500,000 inhabitants	9	23.7%
City over 500,000 inhabitants	2	5.2%

## Data Availability

The raw data supporting the conclusions of this article will be made available by the authors on request.
